# Combinatorial Use of Chitosan Nanoparticles, Reversine, and Ionising Radiation on Breast Cancer Cells Associated with Mitosis Deregulation

**DOI:** 10.3390/biom9050186

**Published:** 2019-05-12

**Authors:** Sofia Piña Olmos, Roberto Díaz Torres, Eman Elbakrawy, Louise Hughes, Joseph Mckenna, Mark A. Hill, Munira Kadhim, Patricia Ramírez Noguera, Victor M. Bolanos-Garcia

**Affiliations:** 1 Laboratorio de Toxicología Celular L-9, Unidad de Investigación Multidisciplinaria UNAM, FES-Cuautitlán. Campo 4. Carretera Cuautitlán-Teoloyucan Km 2.5, San Sebastián Xhala, Cuautitlán Izcalli, Estado de México 54719, Mexico; spina@comunidad.unam.mx (S.P.O.); diaztorres_r@hotmail.com (R.D.T.); 2 Department of Biological and Medical Sciences, Faculty of Health and Life Sciences, Oxford Brookes University, Gipsy Lane, Headington, Oxford OX3 0BP, UK; eelbakrawy@brookes.ac.uk (E.E.); louise.hughes@oxinst.com (L.H.); josephmckenna@brookes.ac.uk (J.M.); mkadhim@brookes.ac.uk (M.K.); 3 CRUK/MRC Oxford Institute for Radiation Oncology, Department of Oncology, University of Oxford, ORCRB Roosevelt Drive, Oxford OX3 7DQ, UK; mark.hill@oncology.ox.ac.uk

**Keywords:** nanoparticles, spindle-assembly checkpoint inhibition, breast cancer, mitosis deregulation, tumour sensitisation

## Abstract

Breast cancer is the most commonly occurring cancer in women worldwide and the second most common cancer overall. The development of new therapies to treat this devastating malignancy is needed urgently. Nanoparticles are one class of nanomaterial with multiple applications in medicine, ranging from their use as drug delivery systems and the promotion of changes in cell morphology to the control of gene transcription. Nanoparticles made of the natural polymer chitosan are easy to produce, have a very low immunogenic profile, and diffuse easily into cells. One hallmark feature of cancer, including breast tumours, is the genome instability caused by defects in the spindle-assembly checkpoint (SAC), the molecular signalling mechanism that ensures the timely and high-fidelity transmission of the genetic material to an offspring. In recent years, the use of nanoparticles to treat cancer cells has gained momentum. This is in part because nanoparticles made of different materials can sensitise cancer cells to chemotherapy and radiotherapy. These advances prompted us to study the potential sensitising effect of chitosan-based nanoparticles on breast cancer cells treated with reversine, which is a small molecule inhibitor of Mps1 and Aurora B that induces premature exit from mitosis, aneuploidy, and cell death, before and after exposure of the cancer cells to X-ray irradiation. Our measurements of metabolic activity as an indicator of cell viability, DNA damage by alkaline comet assay, and immunofluorescence using anti-P-H3 as a mitotic biomarker indicate that chitosan nanoparticles elicit cellular responses that affect mitosis and cell viability and can sensitise breast cancer cells to X-ray radiation (2Gy). We also show that such a sensitisation effect is not caused by direct damage to the DNA by the nanoparticles. Taken together, our data indicates that chitosan nanoparticles have potential application for the treatment of breast cancer as adjunct to radiotherapy.

## 1. Introduction

The high prevalence of breast cancer, the most common type of cancer in women and the second most commonly occurring cancer in the world, represents a global health challenge. Over two million new cases of breast cancer worldwide were reported in 2018 alone [1]. Undoubtedly, the development of new therapeutic strategies to treat breast tumours is of upmost importance. Genome instability and chromosome segregation defects are two defining features of solid tumours, the most aggressive types of cancer, and often associated to defects in the spindle-assembly checkpoint (SAC), which is the molecular signaling mechanism of higher organisms that monitors the proper segregation of the genetic material every time a cell divides [2]. 

Nanoparticles of different composition, including mesoporous silica and metals such as gold, titanium, and molybdenum have been shown to sensitise cancer cells to radiation [3,4,5,6,7], which is an effect that may be due to the accumulation of nanoparticles in tumour neovessels, thus increasing radiation absorption in the tumour tissue without affecting the healthy tissue that surrounds the cancer cells [3]. 

However, the synthesis of these nanoparticles is laborious and expensive, which limits their broader use in the clinic. In contrast, the synthesis of nanoparticles made of the natural polymer chitosan is easy and cheap, with the aggregated value that chitosan nanoparticles are non-immunogenic. Chitosan nanoparticles (CS-NP) can exert multiple influences on cells, such as inducing changes in cell morphology, impacting cellular junctions, impeding motility, and inducing cell gaps [8,9]. 

Moreover, studies on rats have shown that nanoparticles based on chitosan isolated from squid can have an anti-cancer protective effect in liver cells, and that this can be due to the nanoparticles’ antioxidant and anti-lipidemic properties [10,11,12]. Chitosan nanoparticles have gained much attention due their good biocompatibility and biodegradability, and have been approved by the Food and Drug Administration (FDA) for wound-dressing applications [13,14]. The overall shape, size distribution, surface morphology, and surface charge of chitosan nanoparticles dictate the dynamics of their entry and diffusion into the cell [15,16]. Importantly, such physicochemical properties can be easily manipulated to enhance the nanoparticles permeation and retention in tumour sites as well as to use them as drug carriers. 

The inhibition of Mps1 and Aurora B kinase activity results in premature mitosis exit with misattached/unaligned chromosomes, which in turn results in severe chromosome missegregation, aneuploidy, and cell death [2]. Also, nanoparticles made of mesoporous silica and metals can sensitise cancer cells to chemotherapy and radiotherapy. These factors prompted us to investigate the potential sensitisation effect of chitosan nanoparticles to the X-ray irradiation of MCF-7 breast cancer cells treated with the Mps1 and Aurora B inhibitor reversine. Our data show that chitosan nanoparticles by themselves elicit cellular responses that affect mitosis and cell viability and sensitised MCF7 cells to X-ray irradiation, and that this effect is not due to the genotoxicity of the chitosan nanoparticles. These results suggest that this type of nanomaterial can be used to improve the treatment of breast cancer and possibly other types of aggressive tumours as adjunct to radiotherapy. 

## 2. Materials and Methods 

### 2.1. Materials 

Chitosan of low molecular weight and sodium tripolyphosphate were purchased from Sigma Aldrich (Dorset, England). Bis-Tris 12% precast gels were from Thermo Fisher (Walthman, MA, USA). Anti-N-terminal Mps1 antibody (ab135819) was from Abcam (Cambridge, UK). Anti-β-actin antibody was from Wuhan Fine Biotech (Wuhan, China). Anti-rabbit IgG AP-linked and Anti-Phosphohistone-Ser 10-H3 were from Cell Signalling Technology (CST)/NEB (#7054) (Danvers, MA, USA). 

### 2.2. Nanoparticle Synthesis 

Chitosan nanoparticles were manufactured by the ionic gelation method using sodium tripolyphosphate [17,18,19]. For this, 0.3 % (*w*/*v*) of low molecular weight chitosan was prepared in acetic acid 1.0 % *v*/*v*. An equal volume of sodium tripolyphosphate 0.1% (*w*/*v*) was added afterwards. The solution was mixed for 1 h using a magnetic stirrer. The nanoparticles thus formed were filtered through a 0.45-µm membrane (Sarstedt, Nümbrecht, Germany) and stored at 4 °C until use. Fluorescent nanoparticles were prepared as described before [20,21] with the following modifications: as soon as the nanoparticles were formed, a 0.0125 mg/mL rhodamine 123 (Sigma-Aldrich) solution was added to the nanoparticle suspension and left under magnetic stirring for 6 h protected from the light. After this, the nanoparticles were centrifugated at 54000 RCF (Optima XPN-80 ultracentrifuge, Beckman Coulter, Fichtenhain, Germany) to separate dye-labelled and unlabelled nanoparticles. This method relies on the generally accepted assumption that the fluorochrome is absorbed to the nanoparticles’ surface [20].

### 2.3. Nanoparticle Characterisation

Nanoparticles tracking analyses was carried out by dynamic light scattering (DLS) measurements using the Z-view instrument (Particle Metrix, Ammersse, Germany) and by transmission electron microscopy (TEM) using a tungsten filament HV at 100 kV (Hitachi, Tokyo, Japan). For the latter study, a fresh nanoparticle suspension was placed on a copper grid and left undisturbed for 5 min. The excess was removed using filter paper, and the nanoparticle suspension was dried and placed into the microscope for image analysis.

### 2.4. Nanoparticles’ Intracellular Localisation

To investigate the cellular distribution of the CS-NP, they were labelled with rhodamine 123 (Sigma-Aldrich), and the MCF-7 cells were left to grow up on a glass cover slide. Once the cells were attached, CS-NP loaded with rhodamine 123 were added to the cultured cells and incubated for 3 h. As a control, the equivalent amount of rhodamine 123 alone was added to a separate MCF-7 cells culture. After this, both cell cultures were washed twice with phosphate-buffered saline (PBS) (potassium phosphate monobasic 1.05 mM; sodium phosphate dibasic 2.96 mM; sodium chloride 155.17 mM, pH 7.4) for 5 min, fixed with 0.4% paraformaldehyde for 30 min, and washed twice with PBS. The nuclei of fixed cells were stained with 0.5 µM of 4’,6-diamidino-2-phenylindole, dihydrochloride (DAPI) (Fisher Scientific, Loughborough, UK) and mounted on a slide with 10% polyvinyl alcohol (PVA), 2.5% 1,4-diazabicyclo-octane (DABCO), 5% glycerol, and 25 mM of Tris buffer (PVA-DABCO) mounting medium (Sigma-Aldrich). The preparations were kept refrigerated until analysis by laser scanning confocal microscopy (LSCM 880 Zeiss, Berlin, Germany) at 63× magnification.

### 2.5. Cell Culture

One validated cryo-vial sample of the estrogen-dependent human breast cancer cell line Michigan Cancer Foundation-7 (MCF-7) was kindly donated by Dr. Frances Willenbrock (Department of Oncology, University of Oxford, Oxford, UK). Cells were kept in Dulbecco’s Modified Eagle’s Medium (DMEM) Nutrient Mixture F-12 HAM (Sigma-Aldrich), supplemented with 13% heat-inactivated fetal bovine serum (Sigma-Aldrich), 1% Penicillin-Streptomycin (Corning, Flintshire, UK), and 1% L-Glutamine (Gibco, Loughborough, UK) at 37 °C in humidified atmosphere containing 5% CO_2_. Mycoplasma contamination tests were performed every four weeks to ascertain that the cells were mycoplasma-free. 

### 2.6. Cell Irradiation 

MCF-7 cells (1.5 × 10^6^ in total) were seeded on T75 flasks (Sarstedt) and 3 × 10^4^ cells/well seeded on 96-well plates (Grenier Bio-one, Stonehouse, UK). Cells between 70–80% of confluence were treated with CS-NP during 3 h; then, the media was removed and replaced with new media containing reversine 5 µM and incubated for 1.5 h. Previous reports by others have shown that reversine at this relatively high concentration is effective to inhibit the SAC kinases Mps1 and Aurora B in HeLa cells after 24 h of exposure [22,23]. Following sequential treatment with chitosan nanoparticles and reversine, the breast cancer cells were X-ray irradiated at 2 Gray (2 Gy) dose at the Oxford Institute for Radiation Oncology, University of Oxford, using 250-kV X-rays (0.25-mm copper filtration, resulting in a half-value layer of 1.0 mm Cu) at a dose rate of 0.55 Gy/min. The radiation time for all the samples was under 4 min. Dosimetry was performed using Gafchromic EBT3 film (Ashland Advanced Materials, Bridgwater, NJ, USA) in the position of the cells and analysed as previously described [24]. 

### 2.7. Cell Viability Assay

Cell viability after irradiation procedures was tested by the metabolic principle of the active enzymes from the viable cells to reduce the dye resazurin (Fisher Scientific) to resorufin, thereby generating a quantitative measure of viability and cytotoxicity. The dye was added 30 min after irradiation of cells and incubated at 37 °C for 3 h. Then, the fluorescence was measured using a microplate reader (Spectra Max I3, Molecular Devices, Wokingham, UK) with an excitation-emission wavelength of 560/590 nm, respectively.

### 2.8. Comet Assay

The alkaline comet assay [25] was carried out by adopting the protocol originally reported by Singh et al. [26]. After 30 min of X-ray irradiation, the cells were harvested and counted in a Neubauer Chamber using Trypan Blue (Sigma-Aldrich). Viable cells were kept on ice and used to make a 2 × 10^4^ cell suspension. The cells were mixed with 100 µL of 1% (*w*/*v*) low melting point agarose and spread on a frosted microscope slide precoated with 1% (*w*/*v*) normal melting point agarose. A coverslip was used on the smear and the slides were left to solidify for 10 min on ice. The coverslip was removed, and the slides were immersed on fresh cold lysis buffer (2.5 M of NaCl, 100 mM of ethylenediaminetetraacetic acid (EDTA), 10 mM of Tris, 1% dimethyl sulfoxide (DMSO), and 1% Triton X-100 pH10) overnight at 4 °C. The next day, comet slides were placed on a horizontal tank containing electrophoresis buffer (300 mM of NaOH, 1 mM of EDTA pH 13) and left undisturbed for 20 min to allow DNA unwinding. After this, electrophoresis was carried out at 19 V for 30 min. Then, the slides were neutralised with cold neutralisation buffer (0.4 M of Tris-HCl pH 7.5), rinsed with distilled water, and stained with Diamond Nuclei Acid Stain (Promega, Wisconsin, USA). The slides were dried at room temperature, protected from light, and scored using the Comet Assay IV software (Perceptive Instruments, Staffordshire, UK) coupled to an epifluorescent microscope (Axio Scope A 1, Zeiss) with 40× objective magnification.

### 2.9. Mitotic Analysis and Western Blot

Cells after 30 min of X-ray irradiation were washed two times with cold PBS and fixed with 4% paraformaldehyde and 0.1% Triton X-100 during 30 min at room temperature. Then, the cells were washed three times with cold PBS and incubated overnight at 4 °C after the addition of 50 μL/well of a 1:500 dilution of anti-phospho-histone H3 (Cell Signalling Technology, Hertfordshire, UK). After this, the antibody was removed, and the plates washed with PBS. Then, 4 μM of Hoechst 33342 was added, and the plates were scored using an imaging cytometer (Celigo, Nexcelom Bioscience, Manchester, UK). After SDS-PAGE in 12% Bis-Tris precast gels using MOPS-SDS as the running buffer, Western blot analysis was carried out using the primary anti-Mps1 antibody at 1:500 dilution and the secondary anti-rabbit IgG Alkaline Phosphatase (AP)-linked antibody at 1:1000 dilution following a standard protocol. The protein β-actin was used as the loading control and detected using an anti-β-actin antibody. 

### 2.10. Statistical Analyses 

All the experiments were conducted by triplicate and the data were analysed using the Graph Pad Prism 8 software (Northside, San Diego, CA, USA). Cell viability and mitotic index data were assessed by analyses of variance (one-way ANOVA) and multiple means comparisons (*p* < 0.0001) according to the Tukey´s (also known as the honestly significant difference, HSD) test (*p* < 0.05). This type of statistical analysis enables the calculation of minimal differences between means from pairwise comparisons of data groups, where means differing by more than the HSD value indicate a significant difference. For comet assays, the Kruskal–Wallis test was used followed by Dunns (*p* < 0.05) pairs of means comparison.

## 3. Results

### 3.1. Physicochemical Characterisation of Chitosan Nanoparticles 

Initially, the key physicochemical properties of CS-NP, including the nanoparticles concentration, size distribution, and zeta potential, were determined. As Table 1 shows, the CS-NP exhibited a positively charged surface, as reported by the Z potential value, with an average size (hydrodynamic diameter) of around 200 nm. 

### 3.2. Nanoparticles Morphology

**Figure 1:**
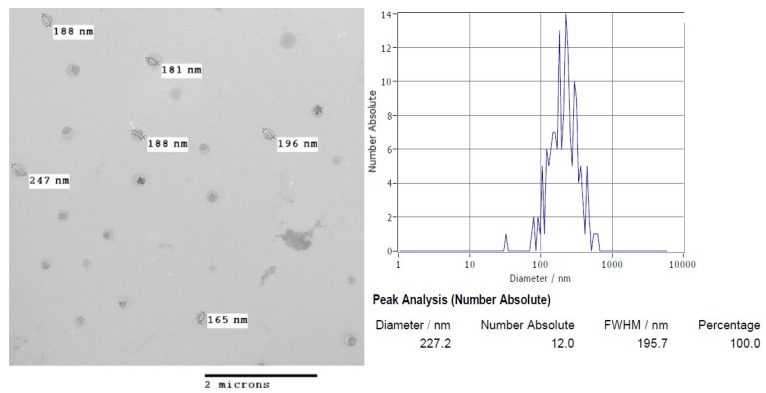
Left, transmission electron microscopy (TEM) image of CS-NP and right, size distribution plot based on nanoparticle tracking analysis.

In an attempt to gain direct insight into the morphology of the chitosan-based nanomaterial, the chitosan nanoparticles were analysed by TEM. Transmission electron microscopy images of CS-NP revealed that the ionotropic gelation method [17,18,19] used for the synthesis of CS-NP was adequate to produce spherical nanoparticles. This class of nanoparticles had spherical morphology and an average size distribution around 200 nm as estimated by nanoparticle tracking analysis from dynamic light scattering (DLS) measurements (see Figure 1 for details). 

### 3.3. Nanoparticles Cellular Localisation

**Figure 2:**
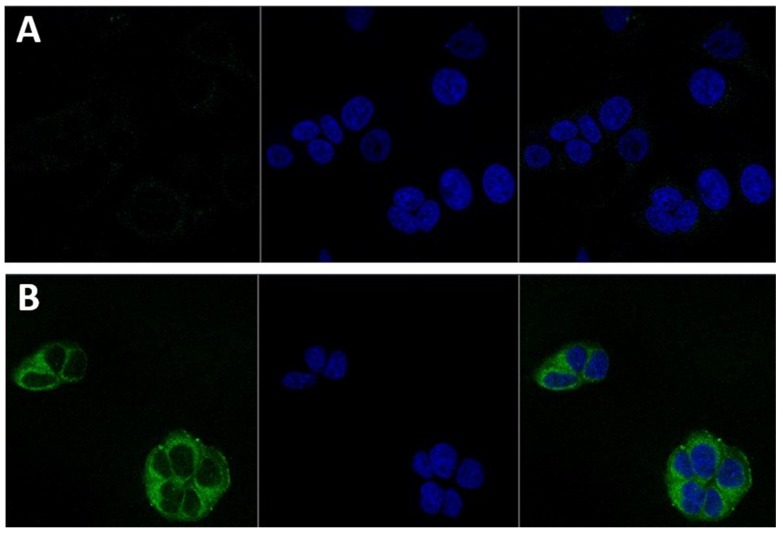
Intracellular localisation of CS-NP in MCF-7 cells as determined by light scattering confocal microscopy. (**A**) Cells with rhodamine 123 alone. (**B**) Cells after three hours of exposure to CS-NP. In both rows, the left panel shows the cytoplasmic distribution of the CS-NP (green); the middle panel shows the 4′,6-diamidino-2-phenylindole (DAPI)-stained nuclei, and the right panel shows the merge image. All the images were taken at 63× magnification.

Since the subcellular localisation of nanoparticles can vary depending on their chemical composition and surface morphology, the subcellular localisation of the CS-NP in MCF-7 cells was investigated by confocal microscopy. To this aim, chitosan nanoparticles were labelled with the fluorescent marker rhodamine 123. The physicochemical parameters of CS-NP did not change with loading the dye (see Table 1 for details). As shown in Figure 2, the CS-NP were easily incorporated into the breast cancer cells and readily distributed in the cytoplasm after three hours of exposure. A wide distribution in the cytoplasm of chitosan nanoparticles loaded with rhodamine 123 has also been observed in olfactory ensheathing cells [20]. After three hours of exposure, CS-NP were not detected in the nuclei. Figure 2 also shows that CS-NP did not influence the overall morphology of MCF-7 cells. 

### 3.4. Cell Viability Assay 

**Figure 3:**
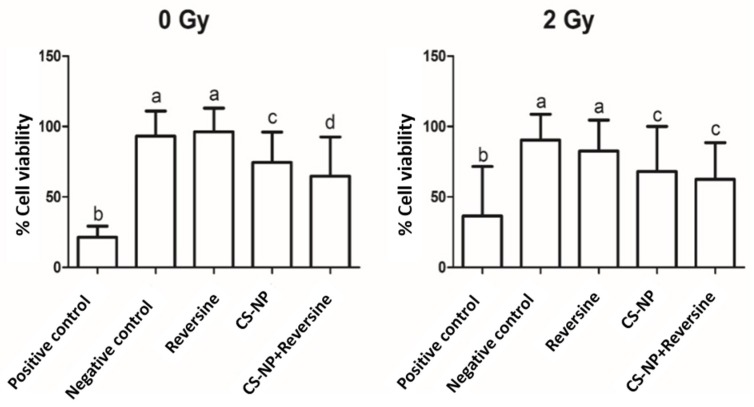
Effect of ionising radiation (2 Gy) on MCF-7 cell viability. Non-irradiated (0 Gy) MCF-7 cells were used as negative control, whereas MCF-7 cells treated with 100 µM of H_2_O_2_ to induce DNA damage following X-ray irradiation were used as positive control. The bars represent the mean value of three independent experiments, and the lines represent the standard deviation. Bars with same letter (a, b, c, d) are not significantly different (Tukey > 0.05). Non-irradiated (0 Gy) samples: negative control, 96.2 ± 9.9; positive control, 36.6 ± 7.8; reversine, 93.1 ± 17.5; CS-NP, 74.5 ± 21.6; CS-NP + reversine, 64.7 ± 26.8. X-ray irradiated (2 Gy) samples: negative control, 90.3 ± 18.8; positive control, 21.3 ± 14.9; reversine, 82.5 ± 20.5; CS-NP, 68.0 ± 29.9; CS-NP + reversine, 62.4 ± 27.0.

After determination of the intracellular localisation of CS-NP, we investigated the potential toxicity of CS-NP on the breast cancer cells. To this aim, the viability of MCF-7 cells treated with CS-NP was determined after X-ray irradiation (2 Gy). Non-irradiated (0 Gy) were used as a control. These experiments showed that the MCF-7 cells’ viability was lower in the irradiated cells compared to the control group. In cells treated with CS-NP + reversine, a similar decrease of cell viability was observed for both non-radiated and X-ray irradiated cells (Figure 3). Reversine inhibits the SAC kinases Mps1 and Aurora B in a dose-dependent manner [23]. Since there was not a significant effect on cell viability in non-radiated (0 Gy) cells compared to the negative control and only a slight effect on cell viability on X-ray irradiated (2 Gy) cells in breast cancer cells that were treated with reversine for only 1.5 h, it is assumed that the observed effect on cell viability is largely due to the CS-NP themselves. Taken together, the data indicate that CS-NP sensitised MCF-7 cells to biological radiation. Since metal-containing nanoparticles have been reported to abrogate DNA repair and cause the extensive damage of telomeres in MCF-7 cells [27], we investigated whether the sensitising effect of the CS-NP was due to the alteration of DNA stability. To address this question, comet assays were carried out in non-irradiated and X-ray irradiated cells. The results of these studies are described below. 

### 3.5. DNA Damage by Comet Assay

**Figure 4:**
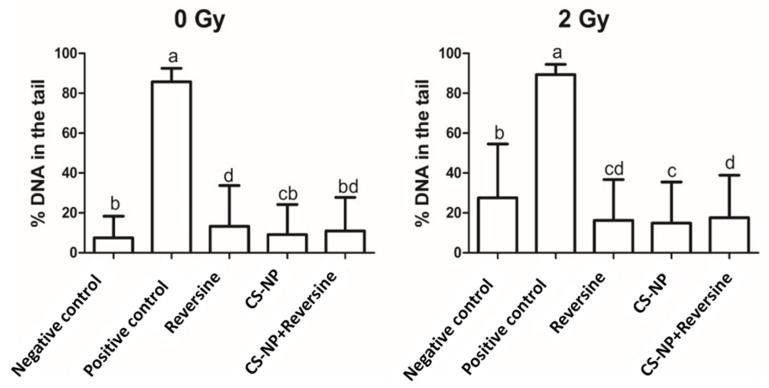
Percentage of DNA damage in non-irradiated (0 Gy) and X-ray irradiated (2 Gy) MCF-7 cells as measured by the alkaline comet assay. H_2_O_2_ at 100 µM was used as the positive control. The bars represent the mean value and the lines represent the standard deviation. Bars with same letter (a, b, c, d) are not significantly different (Dunns > 0.05). The average values for non-irradiated (0 Gy) samples: negative control, 7.4 ± 0.30; positive control, 85.7 ± 6.7 reversine, 13.2 ± 0.59; CS-NP, 9.2 ± 0.43; CS-NP + reversine, 11.0 ± 0.48. Average values for X-ray irradiated (2 Gy) samples: negative control, 27.6 ± 0.77; positive control, 89.3 ± 0.14; reversine, 16.2 ± 0.59; CS-NP, 14.9 ± 0.59; CS-NP + reversine, 17.6 ± 0.61.

**Figure 5:**
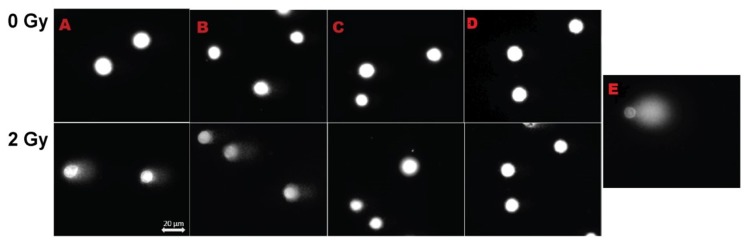
Representative images of the cells assayed by the comet assay. The nuclei was stained with diamond nuclei acid stain. Above, non-irradiated cells. Below, irradiated cells where A = negative control; B = cells treated with reversine, 5 µM; C = cells treated with CS-NP; D = cells treated with CS-NP + reversine, 5 µM; E = positive control, consisting of cells treated with 100 µM of H_2_O_2_. All the images were taken at 40× magnification.

The percentage of DNA damage in non-irradiated (0 Gy) and X-ray irradiated (2 Gy) MCF-7 cells, as determined by the alkaline comet assay, is shown in Figure 4. As anticipated, in both non-irradiated and irradiated cells, the extent of DNA damage was much lower than in the positive control H_2_O_2_ at 100 µM: 85.7 ± 6.7 (0 Gy) and 89.3 ± 0.14 for the irradiated (2 Gy) cells. In non-irradiated (0 Gy) MCF-7 cells, the extent of DNA damage was as follows: CS-NP, 9.2 ± 0.43; reversine, 13.2 ± 0.59; CS-NP + reversine, 11.0 ± 0.48. A slight increase of DNA damage was observed when the cells were irradiated (2 Gy): CS-NP, 14.9 ± 0.59; reversine, 16.2 ± 0.59; CS-NP + reversine, 17.6 ± 0.61. Such effect was clearer for the negative control, were the percentage of DNA in the tail was higher in the irradiated cells (0 Gy, 7.4 ± 0.30; 2 Gy, 27.6 ± 0.77). Then, we used the comet assay to quantify DNA breaks associated with the direct damage to the cells induced by radiation (Figure 5). We selected the comet assay because it is generally recognised as one of the most sensitive methods for analysing and quantifying DNA damage in individual eukaryotic cells. Taken together, the data presented in Figure 4 and Figure 5 indicate that the observed sensitisation of MCF-7 cells by the CS-NP was not directly related to genotoxicity. This is in stark contrast to the cellular effects reported in other types of nanoparticles, where genotoxicity resulting from DNA oxidative lesions has been observed [28]. 

### 3.6. Mitotic Index

**Figure 6:**
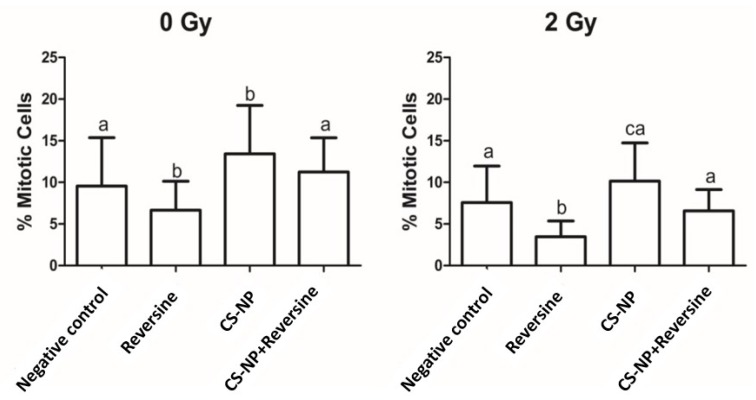
Mitotic index of non-irradiated (0 Gy) and irradiated (2 Gy X-rays) MCF-7 cells. The bars represent the mean values, whereas the lines indicate the standard deviation. Bars with same letter (a, b, c, d) are not significantly different (Tukey > 0.05). Mean values of non-irradiated (0 Gy) samples: negative control, 9.6 ± 5.8; reversine, 6.7 ± 3.4; CS-NP, 13.4 ± 5.8; CS-NP+ reversine, 11.24 ± 4.1. Mean values of X-ray irradiated (2 Gy) samples: negative control, 7.79 ± 4.4; reversine, 3.5 ± 1.8; CS-NP, 10.1 ± 4.5; CS-NP + reversine, 6.7 ± 2.5.

The ATP-binding competitor reversine is known to cause premature exit from mitosis in HeLa, U2OS, and retinal pigment epithelial cells during an unperturbed mitosis and to inhibit the colony formation of human acute myeloid leukemia cells [22,23]. Reversine was first reported as an inhibitor of Aurora B kinase that abolished the Aurora B-dependent phosphorylation of histone H3 [22]. In an attempt to establish whether CS-NP can synergise chemotherapy based on SAC kinases (Mps1 and Aurora kinase B) inhibition by reversine, the number of mitotic cells was estimated by cytometry analysis. Our study shows that in MCF-7 cells treated with reversine, the percentage of cells in mitosis was much lower than in the negative control in both non-irradiated and X-ray irradiated cells (Figure 6). Moreover, the use of an anti-phospho-histone H3 antibody revealed that in non-irradiated and X-ray irradiated cells, the number of MCF-7 cells in mitosis following treatment with CS-NP alone was consistently higher (0 Gy, 13.4 ± 5.8; 2 Gy, 10.1 ± 4.5) than in cells exposed to reversine alone (0 Gy, 6.7 ± 3.4; 2 Gy, 3.5 ± 1.8) and slightly higher to that of cells treated with CS-NP + reversine (0 Gy, 11.24 ± 4.1; 2 Gy, 6.7 ± 2.5). Whether this behaviour indicates mitosis rescue by the CS-NP is uncertain, and is an interesting aspect that warrant further investigations (Figure 6). What is clear from our study is that the combined use of CS-NP + reversine followed by X-ray irradiation (2 Gy) does not lead to a synergistic response in the treated MCF-7 cells, and that the sensitisation effect to X-ray irradiation is due only to the exposure to CS-NP. 

### 3.7. Mps1 Expression

At least in cultured cells, amorphous silica and certain metal-containing nanoparticles can induce genotoxic, cytotoxic, and changes in transcriptomic responses, including the expression of genes related to oxidative stress responses, protein synthesis, and degradative enzymes [29,30,31]. Since Mps1 is a SAC kinase amplified in MCF-7 breast cancer cells [32], we investigated if exposure to the CS-NP before and after ionising radiation had any effect on Mps1 kinase expression levels. Western blot analysis of sodium dodecyl sulfate-polyacrylamide gel electrophoresis (SDS-PAGE) (Supplementary Figure S1) showed that the treatment of MCF-7 cells with CS-NP, reversine, and CS-NP + reversine followed by X-ray irradiation (2 Gy) did not affect the expression of Mps1 kinase in MCF-7 cells. A similar situation was observed in non-irradiated cells. In these studies, β-actin was used as the loading control (Supplementary Figure S1). 

## 4. Discussion

One important problem in clinical practice is that X-ray irradiation of tumour cells can also induced damage to the normal tissue. This situation has boosted the development of novel approaches, including the use of nanotechnology to induce sensitisation to radiation on cancer cells [3]. In this work, we evaluated the use of chitosan nanoparticles to sensitise MCF-7 cells to X-ray irradiation. Characterisation of the CS-NP (show in Table 1 and Figure 1) included determination of the CS-NP size, shape, surface morphology, and surface charge. These are fundamental properties that influence the nanoparticles’ interaction with biomolecules and the uptake mechanisms used by the cells, which include the opening of tight junctions [6] and clathrin and caveolae pathways [9]. Although the study of the precise uptake mechanism of chitosan-based nanoparticles was not addressed in this work, it is important to note that the CS-NP were detected inside the cells after three hours of exposure, and that they were evenly distributed through the cytoplasm (Figure 2), arguing in favour of their use to target cancer cells. It is worth noting that independent studies by other researchers have shown that titania NP doped with rare earth elements is an effective strategy to cause the radiosensitisation of tumour cells [7,33]. Whether the radiosensitisation efficiency and/or stability within the cells of CS-NP is higher than that of metallic nanoparticles, including rare earth elements-doped titania NP, remains to be established. 

Earlier studies on CS-NP showed that high molecular weight chitosan is cytotoxic [34]. However, independent reports by others found no evidence of cytotoxicity of chitosan nanoparticles to MCF-7 cells following exposure for 6 h and 24 h, even at high concentrations of chitosan [31]. Our data is in agreement with the former reports, as it shows that in MCF-7 cells treated with CS-NP, the nanomaterial has a mild negative effect on the viability of MCF-7 breast cancer cells, and that such effect is not due to the induction of DNA damage (Figure 3, Figure 4 and Figure 5). Although producing nanoparticles of different sizes is not relevant to this study, it is worth mentioning that CS-NP of a narrower size distribution can be achieved through a combination of changes in the method of nanoparticles synthesis reported here together with the separation of CS-NP of various dimensions using size exclusion chromatography. It would be interesting to explore if CS-NP of narrower size distribution can enhance their permeation across the cell membrane and/or their retention in tumour sites.

It is worth noting that in mitotic index determination based on the quantification of P-Ser(10)-H3, which is a well-established mitosis marker and Aurora kinase B substrate, the number of mitotic cells treated with CS-NP + reversine was higher than that observed in cells exposed to reversine alone (Figure 6), suggesting a possible rescue of the SAC. Such scenario would argue against the development of a therapeutic strategy aiming to induce premature mitosis exit. However, in our hands, the potential rescue of the SAC in the treated MCF-7 cells was not paralleled by an improvement in cellular viability. Further combinatorial studies using different dose levels of CS-NP and reversine at extended exposure times are required to clarify the apparent SAC rescue induced by CS-NP. Furthermore, in the light of our current understanding of the mechanism of action of SAC inhibitors, targeting mitosis exit inhibition rather than the induction of premature mitotic exit may be advantageous to reduced cytotoxic side effects [35]. This may be particularly effective for mitosis exit inhibitors used in combination with vinca alkaloids (vinblastine, vincristine, vindesine, vinflunine, vinorelbine) and taxanes (docetaxel, taxol), which are two chemical classes of drugs that through different mechanisms of action, induce mitotic arrest by interfering with microtubule assembly, thus promoting genome instability above the threshold required to kill cancer cells [36]. It will be important to assess to what extent CS-NP can sensitise triple negative breast cancer and other types of tumours of poor prognosis to combinations of small size inhibitors of mitosis together with microtubule poisons when used alone and in conjunction with subsequent X-ray irradiation.

## 5. Conclusions

Chitosan nanoparticles constitute a nanomaterial that has the potential to be used as adjunct to radiotherapy given their property to radiosensitise MCF-7 breast tumour cells, possibly as a result of their accumulation (passive or targeted) in the cancer cells. Future work will seek to identify the cell signalling pathway(s) implicated in the sensitisation to the radiation of chitosan nanoparticles, which should provide new molecular insight of cancer cells’ sensitisation to X-ray radiation. This new knowledge may in turn open up new opportunities for the adequate stratification of breast cancer patients in clinical trials, leading to better therapeutic strategies for the treatment of this disease. 

## Figures and Tables

**Table 1:** Physicochemical properties of chitosan nanoparticles (CS-NP). The data corresponds to the average of five independent batches.

Nanoparticles	Physicochemical Properties		
	Z-Potential (z-Average) mV ± SD	Size (Hydrodynamic Ratio) nm ± SD	Concentration Particles/mL ± SD
CS-NP	29.6 ± 9.5	224 ± 31	5.9 × 10^10^ ± 2 × 10^10^
CS-NP [R123]	38 ± 0.16	227 ± 97	4.3 × 10^7^ ± 2 × 10^10^

## Supplementary Materials

The following are available online at https://www.mdpi.com/2218-273X/9/5/186/s1, Figure S1: Western blot of the different samples studied using an anti-N-terminal Mps1 antibody. The abbreviations show here are the same as those described in the main text.
