# Supplementary material for: Combinatorial Use of Chitosan Nanoparticles, Reversine, and Ionising Radiation on Breast Cancer Cells Associated with Mitosis Deregulation
Source: Biomolecules. 2019 May 12;9(5):186. doi: 10.3390/biom9050186 (PMC6571805; doi:10.3390/biom9050186)
Supplement: Supplementary file 1 [file biomolecules-09-00186-s001.pdf]

Supplementary

Combinatorial Use of Chitosan Nanoparticles, Reversine, and Ionising Radiation on Breast Cancer Cells Associated with Mitosis Deregulation

Sofia Piña Olmos [1,2], Roberto Díaz Torres [1], Eman Elbakrawy [2], Louise Hughes [2], Joseph Mckenna [2], Mark A. Hill [3], Munira Kadhim [2], Patricia Ramírez Noguera [1,*] and Victor M. Bolanos-Garcia [2,*]

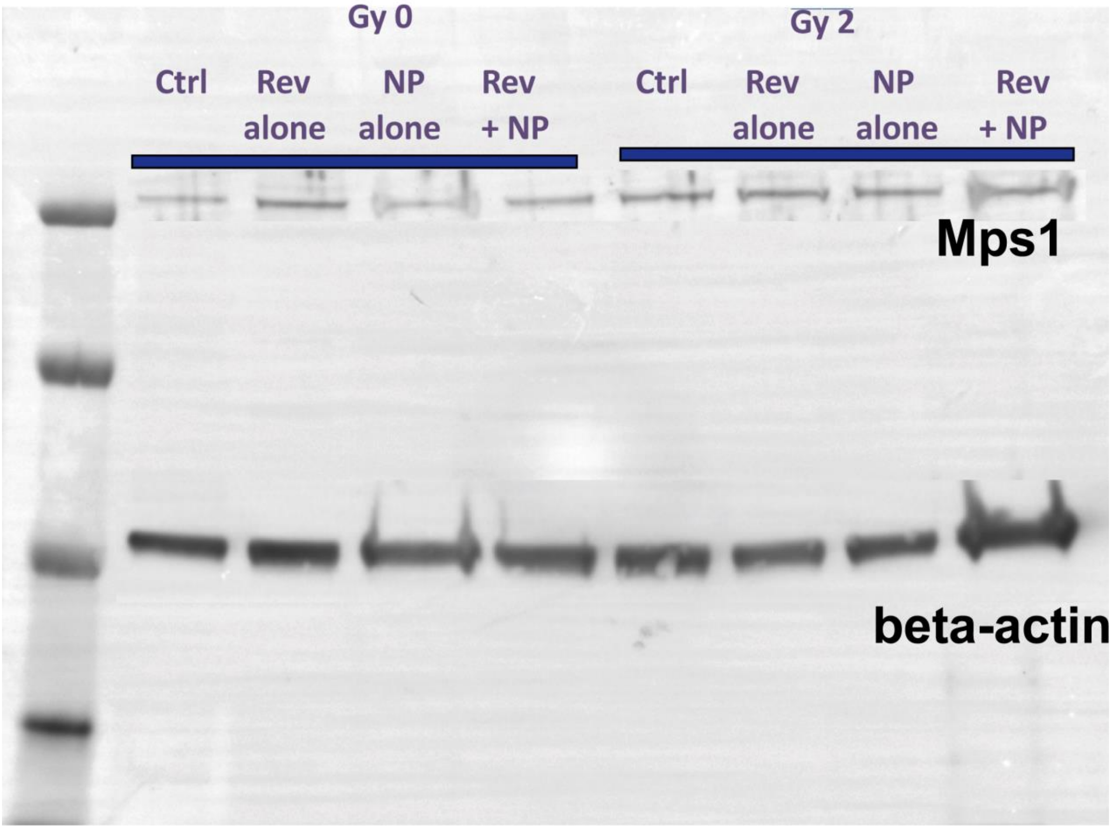

Figure S1. Western blot of the different samples studied using an anti-N-terminal Mps1 antibody. The abbreviations show here are the same as those described in the main text.
